# General Anesthesia-Related Drop in Diastolic Blood Pressure May Impact the Long-Term Outcome in Stroke Patients Undergoing Thrombectomy

**DOI:** 10.3390/jcm11112997

**Published:** 2022-05-25

**Authors:** Alan Abada, Peter Csecsei, Erzsebet Ezer, Gabor Lenzser, Peter Hegyi, Alex Szolics, Akos Merei, Andrea Szentesi, Tihamer Molnar

**Affiliations:** 1 Department of Anesthesiology and Intensive Care, Medical School, University of Pecs, 7624 Pecs, Hungary; alan.abada@gmail.com (A.A.); ezer.erzsebet@pte.hu (E.E.); merei.akos@pte.hu (A.M.); molnar.tihamer@pte.hu (T.M.); 2 Centre for Translational Medicine, Semmelweiss University, 1085 Budapest, Hungary; hegyi.peter@semmelweis-univ.hu (P.H.); a.szentesi@tm-centre.org (A.S.); 3 Department of Neurosurgery, Medical School, University of Pecs, 7624 Pecs, Hungary; lenzser.gabor@pte.hu; 4 Department of Radiology, Örebro University Hospital, 70281 Örebro, Sweden; alexsolich@gmail.com

**Keywords:** ischemic stroke, endovascular treatment, anesthesia, blood pressure, outcome

## Abstract

Background: Several factors affect the efficacy of endovascular thrombectomy (EVT); however, the anesthesia-related factors have not been fully explored. We aimed to identify independent predictors of outcome by analyzing procedural factors based on a multicentric stroke registry. Methods: Data of consecutive patients with acute ischemic stroke (AIS) were extracted from the prospective STAY ALIVE stroke registry. Demographic, clinical, and periprocedural factors including hemodynamic values were analyzed in patients undergoing thrombectomy with either general anesthesia (GA) or conscious sedation (CS). Independent predictors of outcome both at 30 and 90 days based on the modified Rankin Scale (mRS: 0–2 as favorable outcome) were also explored. Results: A total of 199 patients (GA: 76 (38%) vs. CS: 117 (59%); in addition, six patients were converted from CS to GA) were included. The minimum value of systolic, diastolic, and mean arterial pressure was significantly lower in the GA compared to the CS group, and GA was associated with a longer onset to EVT time and a higher drop in all hemodynamic variables (all, *p* < 0.001). A higher drop in diastolic blood pressure (DBP) was even independently associated with a poor 90-day outcome (*p* = 0.024). Conclusion: A GA-related drop in DBP may independently predict a poor long-term outcome in stroke patients undergoing thrombectomy.

## 1. Introduction

Although the efficacy of mechanical thrombectomy in patients with acute ischemic stroke (AIS) has been proven in multiple studies—see Refs. [1,2,3,4,5,6,7,8]—two out of three patients, who receive treatment, are functionally dependent or dead (score on the modified Rankin Scale (mRS > 2)) at 90 days [4]. It is unclear whether the use of general anesthesia (GA) in thrombectomy for AIS interacts with the treatment effect [9]. Since the introduction of mechanical thrombectomy (MT), there has been an ongoing debate regarding the optimal choice of anesthesia for ischemic stroke patients. Recently, a decrease in mean arterial pressure during intervention under GA compared with baseline was found to be associated with a worse outcome [10]. As the impact of anesthesia has not yet been fully explored, the ultimate aim of this study was to identify independent anesthesia-related predictors of the outcome.

An elevated blood pressure level in patients with AIS is common in the emergency phase, suggesting that the collateral perfusion to the penumbra is blood pressure-dependent. However, in the pre-endovascular thrombectomy era, several studies that reported greater values for SBP at the time of stroke presentation were associated with less favorable outcomes [11]. In the MR CLEAN trial, in both endovascular thrombectomy and control patients, the 3-month functional outcome was progressively less favorable when presentation SBP exceeded approximately 130–140 mmHg [12]. Another endovascular thrombectomy study reported that greater SBP values during endovascular thrombectomy were associated with less favorable 3-month functional outcomes [13]. Furthermore, there is also a great body of evidence showing that substantive decreases in blood pressure from values at presentation do not improve outcomes [14]. Moreover, even decreasing blood pressure in the setting of acute stroke is associated with less favorable outcomes [15,16]. The collateral perfusion to the penumbra is not governed by autoregulation [17]. Nevertheless, the influence of detailed hemodynamic values on the outcome in the acute phase of AIS has not been fully evaluated yet. Whereas the guidelines clearly recommend a decrease in blood pressure below 185/110 mmHg in the case of thrombolytic treatment [18], the evidence is less strong for the pre-procedural target of blood pressure in patients eligible for EVT [19].

Instead of using a one-size-fits-all approach, we assumed that an individual approach to blood pressure management would be preferable. An individualized approach may facilitate the avoidance of serious complications such as hemorrhagic transformation or expansion of infarct volume. Whereas the guideline clearly states avoiding hypotension and hypoperfusion in the acute phase of ischemic stroke, there is no recommended cut-off value for blood pressure components [20]. Thus, this study aimed to investigate the relationship between blood pressure components and clinical outcome in patients with AIS after EVT.

## 2. Methods

The study protocol was approved by the Local Ethics Committee at the University of Pécs Medical School and informed consent was obtained from each patient according to the “Good Clinical Practice” (GCP) guidelines (35403-2/2017/EKU) [21].

### 2.1. Study Design and Population

Enrollment in this study was based on a protocol previously published by our research team: the STAY ALIVE Acute Stroke Registry, which is a prospective, ongoing multicenter registry, was designed to build a complete information system for the management of acute ischemic stroke care in the Southern Transdanubian Region, Hungary. Based on census data from 2011, the estimated population of the study area was approximately 1.103.000 inhabitants who lived in area of 16.576 km^2^. Patients outside this area were also admitted on a case-by-case basis. Stroke was defined according to the World Health Organization [22]. All patients with AIS admitted within the time window (4.5 h) and otherwise eligible for intravenous thrombolysis (IVT) received the standard therapy with rt-PA at their local centers. If IVT was ineffective or could not be performed due to contraindication, and the patient had a CTA-confirmed large artery occlusion (ICA, MCA, VA, BA), such patients were referred to our comprehensive stroke center. Clear indications for EVT were the following: (i) demonstration of a large-vessel occlusion by noninvasive vascular imaging; (ii) premorbid mRS (Modified Rankin Scale) score < 3; (iii) ASPECT (Alberta Stroke Program Early CT Score) score > 6 on initial CT scan. The indication of EVT, beyond 6 h of stroke onset, was based on the ASPECT score, because not all primary stroke centers could perform CT perfusion [23]. All ASPECT analyses were independently reviewed by a consultant radiologist from our institution [24].

We retrospectively reviewed our prospectively collected cohort for consecutive patients with AIS who received EVT. However, due to the limited accessibility of anesthesia records, only patients treated in the clinical center of Pécs were included in the statistical analysis.

### 2.2. Anesthesia Protocol

Patients with AIS undergoing an EVT between October 2017 and July 2019 were divided into 2 groups, depending on the type of anesthesia used during the procedure, which was chosen at the discretion of the treating anesthesiologist: patients receiving general anesthesia (GA group) or patients receiving conscious sedation (CS group). Patients converted intraoperatively from CS to GA were considered as part of the GA group. All patients were monitored and managed by anesthesiologists throughout the whole procedure. Propofol (1.5–2.5 mg/kg) was exclusively used as an induction agent, combined with fentanyl and followed by sevoflurane (target: about 1.0 MAC) as maintenance in the GA group. CS included the use of either propofol or midazolam or nalbuphin in a sedative dosage as constant infusion or intravenous bolus at the anesthesiologists discretion. Depth of anesthesia was not monitored by BIS.

GA included induction with propofol, fentanyl, and atracurium, followed by endotracheal intubation and subsequent controlled mechanical ventilation. CS included the use of either propofol, midazolam, or nalbuphin in a sedative dosage as constant infusion or intravenous bolus at the anesthesiologist’s discretion. Furthermore, fentanyl was used in most cases as an analgetic.

Patient monitoring consisted of continuous ECG, pulse oximetry, capnography (end-tidal CO_2_), and invasive blood pressure via arterial catheter [25]. All data were recorded on the anesthetic chart and were uploaded in a blinded manner into the registry dataset. All anesthetic records were hand-written, paper-based protocols.

### 2.3. Data Collection and Outcome Measures

Demography and patient history data (age, sex, preexisting conditions, and their treatment), clinical data (symptom onset to needle time, NIHSS, ASPECTS, heart rate, and blood pressure), and laboratory parameters (INR, Glucose, Creatinine, Platelet Count) were collected. Outcome data were assessed by trained personnel via telephone follow-up. Outcome measures were determined by modified Rankin scale (mRS) after 30 and 90 days. A favorable outcome was defined as mRS 0–2, and a poor outcome as mRS 3–6.

Anesthetic data were collected from the start of anesthesia till the end of the procedure. Extracted data included the type of induction and maintenance, the drugs used, respectively, and hemodynamic values. Blood pressure was measured either non-invasively or invasively. In the case of invasive measurement, these values were preferably used since they are more accurate and readily available.

Hemodynamic values were manually recorded in the anesthetic report every 5 min and manually evaluated. Maximal systolic blood pressure (SBPmax) was defined as the highest periprocedural systolic blood pressure, and minimal systolic blood pressure (SBPmin) as the lowest periprocedural systolic pressure. The systolic blood pressure difference (SBPdiff) was calculated based on the difference between SBPmax and SBPmin. The diastolic blood pressure parameters (maximal diastolic blood pressure (DBPmax), minimal diastolic blood pressure (DBPmin), diastolic blood pressure difference (DBPdiff)) were collected and calculated accordingly. Mean arterial pressure (MAP) was calculated as DBP + 1/3 (SBP-DBP), with MAPmax being the highest MAP, MAPmin being the lowest MAP, and MAPdiff being the difference between these two parameters. Pulse pressure was calculated as the difference between the systolic and diastolic blood pressure, with PPmax being the largest difference between SBP and DBP, PPmin being the lowest difference, and PPdiff being the difference between PPmax and PPmin.

### 2.4. Statistical Analysis

Data were evaluated using the SPSS software package (Version 19.0, IBM Corp., Armonk, NY, USA). The Kolmogorov–Smirnov test was applied to check for normality. Chi-square test for categorical data and Student *t* test for continuous data were used for analysis of demographic and clinical factors. Nonparametric Mann–Whitney U test was used for non-normally distributed values. Data were presented as median and interquartile range (25th–75th percentiles), as well as mean ± SD. A binary logistic regression was used to predict favorable 30- and 90-day outcome, adjusting for age, sex, NIHSS, type of anesthesia, and hemodynamic values. The ideal cut-off value with the best sensitivity and specificity of hemodynamic parameters was determined based on the area under the curve (AUC) using ROC analysis. Correlation analysis was performed by calculating the Spearman correlation coefficient (rho). A *p* value < 0.05 was considered statistically significant. In case of missing data, imputation was not used. All anesthetic data mentioned in the “Data Collection and Outcome Measures” section were accounted for.

## 3. Results

A total of 199 patients were enrolled between October 2017 and July 2019. The mean age was 69 ± 12 years, and 90 patients of the total population were male (45.2%). There was no difference in age and sex distribution in the groups (Table 1). Preexisting conditions (atrial fibrillation, dyslipidemia, diabetes mellitus, hypertension) were also equally distributed in the two groups. The prevalence of hypertension was similar and comparable between the GA (52/75, 70%) and CS 73/109 (67%) groups. Importantly, significantly more female patients presented with prior hypertension (GA group: *n* = 32 vs. 20, *p* = 0.029 and CS group: 46 vs. 27, *p* = 0.017).

In 76 patients (38.2%), GA was the mode of anesthesia, while six more patients (3.0%) had their intervention started under CS, but were later converted into GA during the procedure. The NIHSS before thrombectomy (CS: 11 (IQR:7–16) vs. GA: 14 (IQR: 11–18); *p* = 0.009), after thrombectomy (7 (IQR 2–12) vs. 10.5 (IQR 5–15); *p* = 0.008), after 24 and 72 h (6 (IQR: 1–12) vs. 9 (IQR: 5–14), *p* = 0.003; 5, IQR 0.75–10.25 vs. 8, IQR 4.5–13, *p* = 0.002) were significantly different. The door to recanalization time was on average 19 min longer in the GA group (CS: 61 ± 20 vs. GA: 80 ± 36; *p* = 0.004). There was no difference in hemodynamic parameters on admission (SBP, mmHg: 148 ± 28 vs. 149 ± 25; *p* = 0.405; DBP, mmHg: 84 ± 15 vs. 85 ± 15; *p* = 0.400). However, significant intra-interventional differences in hemodynamics were observed between both techniques. While the maximal blood pressure values (mmHg) have not shown statistical difference (SBPmax: 166 ± 25 vs. 163 ± 23; *p* = 0.538; DBPmax: 92 ± 15 vs. 92 ± 13; *p* = 0.800; MAPmax: 116 ± 16 vs. 115 ± 14; *p* = 0.540; PPmax: 78 ± 20 vs. 78 ± 20; *p* = 0.894), the minimal values were all significantly higher in the CS group than in the GA group (SBPmin: 128 ± 23 vs. 105 ± 25, *p* < 0.001, DBPmin: 70 ± 13 vs. 59 ± 16, *p* < 0.001; MAPmin: 91 ± 15 vs. 75 ± 19; *p* < 0.001; PPmin: 52 ± 15 vs. 40 ± 12; *p* < 0.001). Comparing the CS vs. GA groups, the difference between maximal and minimal pressure values (maximal drop in systolic, diastolic, mean arterial, and pulse pressure, respectively) was significantly lower in patients treated under conscious sedation (SBPdiff: 38 ± 20 vs. 58 ± 28; *p* < 0.001; DBPdiff: 22 ± 12 vs. 33 ± 19; *p* < 0.001; MAPdiff: 25 ± 14 vs. 39 ± 17; *p* < 0.001; PPdiff: 26 ± 14 vs. 37 ± 19; *p* < 0.001), suggesting a favorable intraprocedural hemodynamic state in the CS group.

Significant differences in terms of clinical outcome dependent on DPBdiff were found in patients between both groups: mRS 30 days: 0 (*p* = 0.05), 5 (*p* = 0.034); mRS 90 days: 0 (*p* = 0.019), 4 (*p* = 0.036), and 6 (*p* = 0.020).

While the only independent predictors of 30-day outcome were age and NIHSS at 24 h post-stroke, the latter remained an independent predictor of the 90-day outcome too. Furthermore, the maximal difference in periprocedural diastolic blood pressure (DBPdiff) was also found to be an independent predictor of such long-term outcome (Table 2) (OR: 0.961, 95% CI: 0.93–0.99, *p* = 0.024).

Based on ROC analysis in the total population, the maximal drop of DBP ≥ 27.5 mmHg as a cut-off value predicted a poor 90-day outcome with a sensitivity of 60% and a specificity of 70% (Area: 0.65, 95% Confidence Interval: 0.570–0.736, *p* < 0.001). However, if the same analysis was performed in the GA group, DBPdiff ≥ 32.5 mmHg as a cut-off value predicted a poor 90-day outcome with a sensitivity of 65% and a specificity of 72% (Area: 0.71, 95% Confidence Interval: 0.590–0.821, *p* = 0.002).

**Figure 1:**
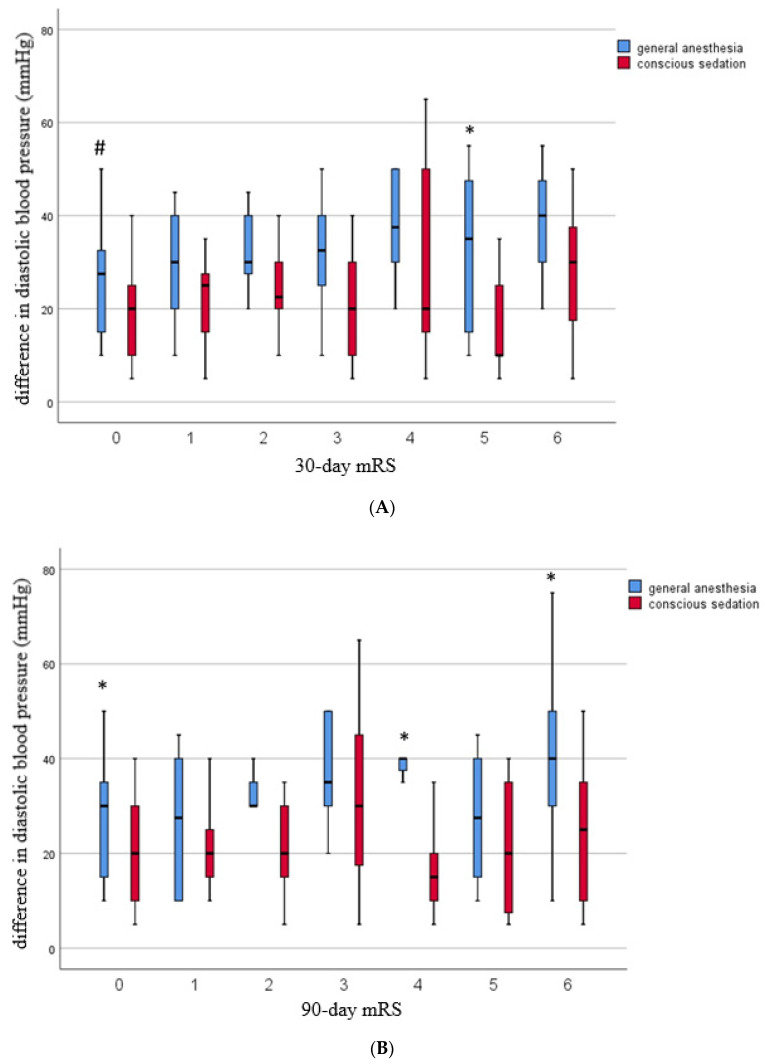
Periprocedural diastolic blood pressure difference compared in the general anesthesia versus conscious sedation related to clinical outcome based on modified Rankin scale (mRS) after 30 days (**A**) and 90 days (**B**), respectively. (**A**) Diagram of the maximal difference in diastolic blood pressure (DBPdiff) during the intervention related to the clinical outcome based on modified Rankin scale after 30 days and the type of anesthesia, respectively (#: *p* = 0.05; *: *p* < 0.05). (**B**) Diagram of the maximal difference in diastolic blood pressure (DBPdiff) during the intervention related to the clinical outcome based on modified Rankin scale after 90 days and the type of anesthesia, respectively (*: *p* < 0.05).

The 30-day and 90-day mRS scores and their respective DBP difference values are displayed in Figure 1A,B, respectively.

## 4. Discussion

We analyzed the data of 199 patients who underwent mechanical thrombectomy (alone or in combination with intravenous thrombolysis) under general anesthesia (GA) or conscious sedation (CS). Analyzing the clinical appearance of the patients undergoing EVT, a lower NIHSS after thrombectomy, at 24 h and at 72 h, could be seen in the CS group. However, their baseline NIHSS prior to thrombectomy was also significantly lower. A possible explanation for these findings could be the significantly longer door to revascularization time in the GA group, which could be observed. Other studies show different results regarding the periprocedural times, such as door to groin puncture time, door to revascularization time, or onset to revascularization time, with some conflicting results [26] and other similar results [27]. There is a point to be made, however, that the preprocedural preparation connected to GA could be causing the prolonged door to groin time.

In the analysis of interventional hemodynamic parameters, the maximal values of systolic and diastolic blood pressure (SBPmax, DBPmax), mean arterial pressure (MAPmax), and pulse pressure (PPmax) showed no significant difference. However, the minimal pressures were significantly lower in the GA group compared to the CS group, as can be explained by the systemic cardiovascular effect of anesthetic agents. Comparable findings have been shared in the past by Melinda, J. et al., even though this comparatively older study had its own limitations [28]. Simonsen et al. could also show a significant difference between the minimal MAP and systolic blood pressure, as well as a more commonly occurring periprocedural drop (>20%) in the case of GA, in the GOLIATH study [29]. In contrast, the outcome measurements were shown to be more favorable in the GA group in terms of final infarct volume and mRS after 90 days, most likely due to the higher rate of successful reperfusion. Furthermore, the authors of the study argue that there might be a bias leading to more severe cases being treated under GA in non-randomized trials, which was not the case in the MR-Clean trial. The differences between the maximum and minimum of the interventional hemodynamic parameters (SBPdiff, DBPdiff, MAPdiff, PPdiff) were all significantly lower in the CS group, indicating that conscious sedation may rather preserve hemodynamic stability compared to general anesthesia. These data are concurrent with other studies showing GA to have lower minimal hemodynamic values and also worse outcome measures [30]. According to a recent study, blood pressure reduction before recanalization was found to be associated with a larger infarct volume and worse functional outcome in patients with large-vessel occlusion stroke [15].

Despite the differences in the hemodynamic values, the only independent predictors of the short-term outcome were age and post-procedural NIHSS, suggesting that older patients and patients with a higher NIHSS after thrombectomy have a worse clinical outcome after 30 days. Other studies have shown similar predictors of 30-day clinical outcome [31,32]. Importantly, the maximal difference in periprocedural diastolic blood pressure (DBPdiff) was also found to be an independent predictor of the 90-day outcome, suggesting the role of diastolic blood pressure in long-term outcome. Some limitations also should be mentioned: (i) the concentration of anesthetic agents was not measured; (ii) the hemodynamic values in the post-anesthesia period were not included into the analysis [33].

Regarding outcome measurements, there seems to be an association between the type of anesthesia and clinical outcome, with people in the CS group showing a more favorable outcome. As shown, a higher DBP and a lower difference between maximal and minimal periprocedural diastolic pressures might be associated with a favorable clinical outcome. Patients with a larger drop in periprocedural DBP undergoing thrombectomy under GA showed higher mortality than patients under CS. Nevertheless, this should be validated in a larger prospective trial including more anesthesia and neurointervention-related factors (collateral circulation, thrombo-inflammatory state, complications such as procedural bleeding, aspiration, and post-stroke infection) [24,34,35,36]. Whereas it seems that CS has less hemodynamic implications and possibly provides a faster intervention, there are special circumstances that indicate GA, such as risk of aspiration, low initial GCS, etc. Since these are connected with a worse pre-procedural patient status, there might be a bias among this and other studies, which found a worse outcome in patients undergoing EVT in GA. A recent study, for example, found similar outcome measures for GA and CS with non-significant time differences [37].

This study shows, on one hand, the impact in terms of hemodynamic volatility in patients suffering from AIS treated with GA compared with CS. Furthermore, it may suggest an association of hemodynamic values, the DBPdiff specifically, with long-term clinical outcome and mortality.

However, this study has several limitations: (i) this is a post-hoc analysis, which means that the results should be further investigated by a randomized control trial with the aim to find differences in hemodynamic values between GA and CS; furthermore, since it is an observational study, there might have been a bias towards using GA patients with initially worse clinical appearance (the baseline NIHSS were higher in the GA group than in the CS group); (ii) this is a single-center analysis of a multi-center registry, resulting in possible treatment bias, since the induction and maintenance was left to the anesthesiologist’s discretion; (iii) only 199 patients were enrolled in this study, with only 76 being in the GA subgroup; further studies, ideally randomized controlled trials, should work with larger and more balanced subgroups; (iv) the presence of missing data may have introduced bias into our study; however, in our most important outcome (mRS after 30 and 90 days), data appear to be missing at random; (v) since the method of blood pressure measurement was not predetermined, some patients had invasive and others non-invasive blood pressure measurement, resulting in possible discrepancies and thus not being exactly comparable; (vi) the baseline NIHSS were higher in the GA group than in the CS group; this might explain the lower postprocedural NIHSS in the CS group; (vii) post-procedural hemodynamic parameters were not monitored as part of this study; however, all recruited patients were monitored and managed in a neurosurgical intensive care unit and post-procedural blood pressure was maintained according to current guidelines.

In conclusion, these findings underscore the importance of hemodynamic management during EVT and highlight the need for further investigation of blood pressure management after large-vessel occlusion stroke.

## Figures and Tables

**Table 1:** Demography, clinical, and outcome data.

	General Anesthesia *n* = 82 (41%)	Conscious Sedation *n* = 117 (59%)	*p*-Value
Demographics			
Age, *y*	83 ± 18	58 ± 15	0.183
Male, *n* (%)	34 (41.5%)	56 (47.9%)	0.791
BMI	27 ± 6 (*n* = 48)	27.2 ± 5 (*n* = 78)	0.448
Comorbidities *n* (%)			
Atrial fibrillation, *n* (%)	35/82 (42.7%)	48/106 (45.3%)	0.759
Diabetes mellitus, *n* (%)	20/75 (26.7%)	30/106 (28.3%)	0.808
Clinical presentation			
IVT before EVT	31/82 (37.8%)	35/117 (29.9%)	0.245
NIHSS before thrombectomy,	14 (IQR: 11–18) (*n* = 60)	11 (IQR:7–16) (*n* = 103)	0.009
NIHSS after thrombectomy	10.5 (IQR 5–15) (*n* = 70)	7 (IQR 2–12) (*n* = 107)	0.008
NIHSS at 24 h after onset	9 (IQR: 5–14) (*n* = 73)	6 (IQR: 1–12) (*n* = 111)	0.029
ASPECTS	7.8 ± 0.9 (*n* = 39)	8.1 ± 1.0 (*n* = 60)	0.294
Outcome			
In-hospital death, *n* (%)	16/82 (19.5%)	15/117 (12.8%)	0.200
Good outcome 30 days (mRS 0–2)	30/72 (41.7%)	56/104 (53.8%)	0.143
Good outcome 90 days (mRS 0–2)	34/69 (49.3%)	60/100 (60%)	0.146
Time metrics, minutes			
Onset to recanalization, min	377 ± 144 (*n* = 39)	333 ± 199 (*n* = 45)	0.260
Door to recanalization, min	80 ± 36 (*n* = 45)	61 ± 20 (*n* = 53)	0.004

BMI, body mass index; *n*: number of patients with valid data; IVT, intravenous thrombolysis; EVT, endovascular thrombectomy; NIHSS, National Institute of Health Stroke Scale; ASPECTS, Alberta Stroke Program Early CT Score; mRS, modified Rankin Scale. Data are presented as absolute number (percentage) or mean ± SD. Chi-square test and Student *t* test were used.

**Table 2:** Binary logistic regression analysis for variables independently associated with favorable outcome (mRS ≤ 2) on day 30 and 90.

**30-Day Favorable Outcome**				
**Variables**	**B**	**Odds Ratio**	**95% CI**	***p*-Value**
age	−0.031	0.969	0.94–1.00	0.050
sex	0.022	1.023	0.49–2.10	0.951
NIHSS at 24 h	−0.077	0.926	0.88–0.97	0.004
type of anesthesia	−0.423	0.655	0.31–1.37	0.259
DBPmin	−0.022	0.978	0.95–1.01	0.174
DBPdiff	−0.027	0.973	0.94–1.01	0.107
**90-Day Favorable Outcome**				
age	−0.023	0.977	0.95–1.01	0.152
sex	−0.002	0.998	0.48–2.06	0.995
NIHSS at 24 h	−0.068	0.934	0.89–0.98	0.008
type of anesthesia	−0.218	0.804	0.38–1.69	0.566
DBPmin	−0.018	0.982	0.95–1.01	0.263
DBPdiff	−0.039	0.961	0.93–0.99	0.024

NIHSS, National Institute of Health Stroke Scale; DBPmin, minimum value of diastolic blood pressure during anesthesia; DBPdiff, maximal drop in DBP during anesthesia.

## Data Availability

All relevant data are within the manuscript.
